# Supplementary material for: T. cruzi DNA polymerase beta (Tcpolβ) is phosphorylated in vitro by CK1, CK2 and TcAUK1 leading to the potentiation of its DNA synthesis activity
Source: PLoS Negl Trop Dis. 2021 Jul 14;15(7):e0009588. doi: 10.1371/journal.pntd.0009588 (PMC8312956; doi:10.1371/journal.pntd.0009588)
Supplement: S1 Fig — Sequences were aligned using the ClustalW tool (https://www.genome.jp/tools-bin/clustalw). The conserved active site of the enzyme from different species is indicated. (PDF) [file pntd.0009588.s001.pdf]

```
CLUSTAL O(1.2.4) multiple sequence alignment

                                             ACTIVE SITE
CK1a_S_pombe        -----MSGQNNVVGVHYKVGRRIGEGSFGVIFEGTNLLNNQQVAIKFEPRRSDAPQLRDE    55
CK1a_Rattus         MASSSGSKAEFIVGGKYKLVRKIGSGSFGDIYLAINITNGEEVAVKLESQKARHPQLLYE    60
CK1a_H_sapiens      MASSSGSKAEFIVGGKYKLVRKIGSGSFGDIYLAINITNGEEVAVKLESQKARHPQLLYE    60
CK1a_T_cruzi        ------MSLELRVGNRFRLGQKIGAGSFGEIFRGTNIQTGETVAIKLEQAKTRHPQLAFE    54
CK1a_Leishmania     --MSSALENCLVQGGRFRLGRRIGGGSFGEIFLGVNTQTGEVVAMKVERTRTAHPQLLSE    58
                                 * :::: ::** **** *: . *  ..: **:*.*  ::  ***  *
                                        ACTIVE SITE
CK1a_S_pombe        YRTYKLLAGC---TGIPNVYYFGQEGLHNILVIDLLGPSLEDLLDLCGRKFSVKTVAMAA    112
CK1a_Rattus         SKLYKILQGG---VGIPHIRWYGQGKDYNVLVMDLLGPSLEDLFNFCSRRFTMKTVLMLA    117
CK1a_H_sapiens      SKLYKILQGG---VGIPHIRWYGQEKDYNVLVMDLLGPSLEDLFNFCSRRFTMKTVLMLA    117
CK1a_T_cruzi        ARFYRILNAGGGVVGIPNILFYGVEGEFNVMVMDLLGPSLEDLFSFCDRKLSLKTTLMLA    114
CK1a_Leishmania     SRYYTLLSQGRGAAYMPTIFGYSSEGEFNVMVMELMGLSLEDLHEKCGNRFSLKTTLMLA    118
                     : * :*      . :* :  :.    .*::*::*:* ***** . *..::::**. * *
                                   ACTIVE SITE
CK1a_S_pombe        KQMLARVQSIHEKSLVYRDIKPDNFLIGRPNSKNA-------------------------    147
CK1a_Rattus         DQMISRIEYVHTKNFIHRDIKPDNFLMGIGRHCNK-------------------------    152
CK1a_H_sapiens      DQMISRIEYVHTKNFIHRDIKPDNFLMGIGRHCNKCLESPVGKRKRSMTVSTSQDPSFSG    177
CK1a_T_cruzi        EQMIARIEFVHSKSVIHRDMKPDNFLMGTGKKGHH-------------------------    149
CK1a_Leishmania     DQILWLIELVHSHSVLHRDIKPDNFLMGTGKKGHH-------------------------    153
                    .*::  :: :* :..::**:******:*   .  :
                                        ACTIVE SITE
CK1a_S_pombe        -NMIYVVDFGMVKFYRDPVTKQHIPYREKKNLSGTARYMSINTHLGREQSRRDDLEALGH    206
CK1a_Rattus         ---LFLIDFGLAKKYRDNRTRQHIPYREDKNLTGTARYASINAHLGIEQSRRDDMESLGY    209
CK1a_H_sapiens      LNQLFLIDFGLAKKYRDNRTRQHIPYREDKNLTGTARYASINAHLGIEQSRRDDMESLGY    237
CK1a_T_cruzi        ---VYVVDFGLAKKYRDPRTHQHIPYKEGKSLTGTARYCSINTHLGIEQSRRDDLEGIGY    206
CK1a_Leishmania     ---VYIIDFGLAKKYRDPRTHAHIPYKEGKSLTGTARYCSINTHLGAEQSRRDDMEGIAY    210
                       ::::***:.* ***  *: ****:* *.*:***** ***:*** *******:*.:.:

CK1a_S_pombe        VFMYFLRGSLPWQGLKAATNKQKYERIGEKKQSTPLRELCAGFPEEFYKYMHYARNLAFD    266
CK1a_Rattus         VLMYFNRTSLPWQGLKAATKKQKYEKISEKKMSTPVEVLCKGFPAEFAMYLNYCRGLRFE    269
CK1a_H_sapiens      VLMYFNRTSLPWQGLKAATKKQKYEKISEKKMSTPVEVLCKGFPAEFAMYLNYCRGLRFE    297
CK1a_T_cruzi        ILMYFLRGSLPWQGLPAATKQEKYVAIAKCKMSSSLETLCKGFPAEFAAYLNYTRSLRFE    266
CK1a_Leishmania     LLIYFLRGSLPWQGLRASTKERKYSLIAHAKMSTSVETLCKGLPIELASFLNYSRALRFE    270
                    :::** * ******* *:*::.**  *.. * *: :. ** *:* *:  :::* * * *:

CK1a_S_pombe        ATPDYDYLQGLFSKVLERLNTTEDENFDWNLLNNGKGWQSLKSRNAETENQRSSKPPAPK    326
CK1a_Rattus         EAPDYMYLRQLFRILFRTLNHQYDYTFDWTMLKQKAAQQAASSSGQG---QQAQTPTGF-    325
CK1a_H_sapiens      EAPDYMYLRQLFRILFRTLNHQYDYTFDWTMLKQKAAQQAASSSGQG---QQAQTPTGKQ    354
CK1a_T_cruzi        DKPDYSYLKRLFRELFIREGYHVDYVFDWTLKRIHESLQDEEKELSN---K---------    314
CK1a_Leishmania     DRPDYGYLRSMFRRLFMREGYQEDYVYDWTVRSMHETLTARQRKNAA---KR-----GKK    322
                      *** **: :*  ::   .   *  :**.:            .        :

CK1a_S_pombe        LESKSPALQNHASTQNVVSKRSDYEKPFAEPHLNSASDSAEPNQNSLPNPPTETKATTTV    386
CK1a_Rattus         ------------------------------------------------------------    325
CK1a_H_sapiens      TDKTKSNMKGF-------------------------------------------------    365
CK1a_T_cruzi        ------------------------------------------------------------    314
CK1a_Leishmania     QK----------------------------------------------------------    324


CK1a_S_pombe        PDRSGLATNQPAPVDVHDSSEERVTREQVQNATKETEAPKKKKSFWASILSCCSGSNEDT    446
CK1a_Rattus         ------------------------------------------------------------    325
CK1a_H_sapiens      ------------------------------------------------------------    365
CK1a_T_cruzi        ------------------------------------------------------------    314
CK1a_Leishmania     ------------------------------------------------------------    324
```

Figure S1: Multiple alignment sequence of CK1 orthologous from different species. Sequences were aligned using the ClustalW tool (https://www.genome.jp/tools-bin/clustalw). The conserved active site of the enzyme from different species is indicated.
