# Supplementary material for: T. cruzi DNA polymerase beta (Tcpolβ) is phosphorylated in vitro by CK1, CK2 and TcAUK1 leading to the potentiation of its DNA synthesis activity
Source: PLoS Negl Trop Dis. 2021 Jul 14;15(7):e0009588. doi: 10.1371/journal.pntd.0009588 (PMC8312956; doi:10.1371/journal.pntd.0009588)
Supplement: S2 Fig — Sequences were aligned using the ClustalW tool (https://www.genome.jp/tools-bin/clustalw). The conserved active site of the enzyme from those species is indicated. (PDF) [file pntd.0009588.s002.pdf]

```
CLUSTAL O(1.2.4) multiple sequence alignment


CK2a_S_pombe        -------------------------------------------------------MNQTE     5
CK2a_Rattus         -----------------------------------------------------------M     1
CK2a_H_sapiens      -----------------------------------------------------------M     1
CK2a_T_cruzi        MTDQDSSGRIIIVTRPVPASPRPPENNGDVSHVTTDCKSQKNTGENTVEAKRTLTENEIR    60
CK2a_Leishmania     -----------------------------MANVDAAAHS-ADGGRHGGGGGGSGDESKAK    30

                                                 ACTIVE SITE
CK2a_S_pombe        AAPVVSVSRVYAHVNEEMPREYWDYENMQEVFGY-QDNYEIIRKVGRGKYSEVFEGLNVL    64
CK2a_Rattus         SGPVPSRARVYTDVNTHRPREYWDYESHVVEWGN-QDDYQLVRKLGRGKYSEVFEAINIT    60
CK2a_H_sapiens      SGPVPSRARVYTDVNTHRPREYWDYESHVVEWGN-QDDYQLVRKLGRGKYSEVFEAINIT    60
CK2a_T_cruzi        HHETGVISHPFWFVNNNMPPSYWDYERMTIEYSS-GEPYELIQKIGRGKYSEVFRCRNRI   119
CK2a_Leishmania     KTKPEEYEHPFWYVYRQRGVSYWDYKNARVDFNANLAPYELLQKIGRGKYSEVFRGRNRN    90
                            : :  *  .   .****:     :.     *::::*:*********.  *
                      ACTIVE SITE / CK2β INTERACTION SURFACE
CK2a_S_pombe        NNRKCIIKVLKPVKYKKIKREIKILQNLAGGPNIISLLDIVRDPESKTPSLIFEFVDNID   124
CK2a_Rattus         NNEKVVVKILKPVKKKKIKREIKILENLRGGPNIITLADIVKDPVSRTPALVFEHVNNTD   120
CK2a_H_sapiens      NNEKVVVKILKPVKKKKIKREIKILENLRGGPNIITLADIVKDPVSRTPALVFEHVNNTD   120
CK2a_T_cruzi        NGELCVLKILKPVRLKKIHREISILQNLCGGPNVLRLLDVVSISPEGTPVLVTENLEPAE   179
CK2a_Leishmania     NGCLCVLKLLKPVRYQKILREISILRNLCGGPNVVRLLDVLRDTESQTVVLVTEYVHNPT   150
                    *.   ::*:****: :** ***.**.** ****:: * *::    . *  *: * :.
                                                 ACTIVE SITE
CK2a_S_pombe        -FRTL--YPTLSDYDIRYYLYELLKALDFCHSRGIMHRDVKPHNVIIVHKKRKLRLIDWG   181
CK2a_Rattus         -FKQL--YQTLTDYDIRFYMYEILKALDYCHSMGIMHRDVKPHNVMIDHEHRKLRLIDWG   177
CK2a_H_sapiens      -FKQL--YQTLTDYDIRFYMYEILKALDYCHSMGIMHRDVKPHNVMIDHEHRKLRLIDWG   177
CK2a_T_cruzi        SFRSLMNSGSLSNFDMRYYMYEVLRCLHFAHSHGIFHRDIKPHNIIIDHQRRKLRIADWG   239
CK2a_Leishmania     TLRNLLYSNKLSNFDMRYYLYEILRSLDFAHRRGIFHRDIKPYNVMIDHERKILRVIDWG   210
                     :: *    .*:::*:*:*:**:*:.*.:.*  **:***:**:*::* *::: **: ***
                        ACTIVE SITE / ACTIVATION LOOP
CK2a_S_pombe        LAEFYHAGMEYNVRVASRYFKGPELLVDFREYDYSLDIWSFGVMFAALIFKKEPFFRGRD   241
CK2a_Rattus         LAEFYHPGQEYNVRVASRYFKGPELLVDYQMYDYSLDMWSLGCMLASMIFRKEPFFHGHD   237
CK2a_H_sapiens      LAEFYHPGQEYNVRVASRYFKGPELLVDYQMYDYSLDMWSLGCMLASMIFRKEPFFHGHD   237
CK2a_T_cruzi        LGEYYIHGQAYNVCVGTRNFKAPELLLGLRLYDYSLDIWSVGCILAEMLFRIFPFFRGQN   299
CK2a_Leishmania     LGEYYIHGQALNCGVATRHYKGPELLVGYRHYDYSLDIWCLGCVLAGMLFRSDPFFVGAN   270
                    *.*:*  *   *  *.:* :*.****:. : ******:*..* ::* ::*:  *** * :

CK2a_S_pombe        NYDQLVKIAKVLGTDELFAYVQKYQIVLDRQYDNILGQYP--KRDWY-FVN--RDNRSLA   296
CK2a_Rattus         NYDQLVRIAKVLGTEDLYDYIDKYNIELDPRFNDILGRHS--RKRWERFVH--SENQHLV   293
CK2a_H_sapiens      NYDQLVRIAKVLGTEDLYDYIDKYNIELDPRFNDILGRHS--RKRWERFVH--SENQHLV   293
CK2a_T_cruzi        NEDQLYRILEVIGTEDLTRYARKYDISLPRFLFGSGGLFKRMKKPWYIF--VNDQCESWC   357
CK2a_Leishmania     NEDQLLQIVAVFGTKALYRYLDKYQCRISRVVESSMSALPDEHVDWRRYIKRGSVQESWC   330
                    * *** :*  *:**. *  *  **:  :     .  .     :  *  :       .

CK2a_S_pombe        NDEAIDLLNRLLRYDHQERLTCQEAMAHPYFQVLK-------------------------   331
CK2a_Rattus         SPEALDFLDKLLRYDHQSRLTAREAMEHPYFYTVVKDQARMSSAGMAGGSTPVSSANMMS   353
CK2a_H_sapiens      SPEALDFLDKLLRYDHQSRLTAREAMEHPYFYTVVKDQARMGSSSMPGGSTPVSSANMMS   353
CK2a_T_cruzi        DVHAVDLLDKMLRLDHQERILAWDAMQHPFFDPIRSALREDPQEQYPQ------------   405
CK2a_Leishmania     DATALDLLDKMLQFDHQDRIMAHEAMQHPFFAPVRDALARDSQEHYPVARR---------   381
                    .  *:*:*:::*: ***.*: . :** **:*  :

CK2a_S_pombe        --------------------------------------     331
CK2a_Rattus         GISSVPTPSPLGPLAGSPVIAAANSLGIPVPAAAGAQQ     391
CK2a_H_sapiens      GISSVPTPSPLGPLAGSPVIAAANPLGMPVPAAAGAQQ     391
CK2a_T_cruzi        --------------------------------------     405
CK2a_Leishmania     --------------------------------------     381
```

Figure S2: Multiple sequence alignment of CK2α orthologous from the indicated species. Sequences were aligned using the ClustalW tool (https://www.genome.jp/tools-bin/clustalw). The conserved active site of the enzyme from those species is indicated.
