# Supplementary material for: T. cruzi DNA polymerase beta (Tcpolβ) is phosphorylated in vitro by CK1, CK2 and TcAUK1 leading to the potentiation of its DNA synthesis activity
Source: PLoS Negl Trop Dis. 2021 Jul 14;15(7):e0009588. doi: 10.1371/journal.pntd.0009588 (PMC8312956; doi:10.1371/journal.pntd.0009588)
Supplement: S3 Fig — Sequences were aligned using the ClustalW tool (https://www.genome.jp/tools-bin/clustalw). S. pombe CK2β possess four CK2α target serine residues at the first 14 aminoacid residues at the N-terminus domain of the polypeptide. Those serine residues are underlined. (PDF) [file pntd.0009588.s003.pdf]

```
CLUSTAL O(1.2.4) multiple sequence alignment


CK2b_T_cruzi        MSYPTYTGVFSDDMAAYEDEADEEALPWIVWFCELKSHEFFCVVDREFIDDEFNLTGLST    60
CK2b_Leishmania     -----MDDAYSPENMEYE-YEDEELVSWITWFCDLKGNEFFCMVDREFITDDFNLTGLAP    54
CK2b_S_pombe        ------MQLYS------SESESDDSQYWVDWFLGLKGNEFFCEVDEDFIQDRFNLTGLSH    48
CK2b_Rattus         ------------------MSSSEEVSWISWFCGLRGNEFFCEVDEDYIQDKFNLTGLNE    41
CK2_H_sapiens       ------------------MSSSEEVSWISWFCGLRGNEFFCEVDEDYIQDKFNLTGLNE    41
                                         ..:   *: **  *:.:**** **.::* * ******

CK2b_T_cruzi        MVSFYHYALDLILDLETQNSARLTAEQQRLVESSAETLYGLIHARFITTQRGLKLMEEKF    120
CK2b_Leishmania     IVPFYHYALELILDVESMESDGLTEQQKRLVESSAEILYGLIHARFITTGRGLKLMEEKY    114
CK2b_S_pombe        EVPHYSQSLDLILDVLDPDLPE---EVQDEVEASARHLYGLIHARYILTAQGLYKMLEKY    105
CK2b_Rattus         QVPHYRQALDMILDLEPDEELEDNPNQSDLIEQAAEMLYGLIHARYILTNRGIAQMLEKY    101
CK2_H_sapiens       QVPHYRQALDMILDLEPDEELEDNPNQSDLIEQAAEMLYGLIHARYILTNRGIAQMLEKY    101
                     *  .*  :*::***:   :      : .  :* :*. ********:* * :*:  * **:

CK2b_T_cruzi        AEGEFGRCPRVFCGGQAVLPVGQSDVVRESSVKLYCPKCQDIYYPRSSRHRTLDGAFWGT    180
CK2b_Leishmania     MQGEFGSCPRVFCEGHALLPVGQSDVVRESSVKLFCPRCEDIYHPRSVRHRSLDGAFWGT    174
CK2b_S_pombe        KKCDFGHCPRVLCNGQPMLPVGLSDIAHAKSVKLYCPRCEDVYTPKSQRHASIDGAYFGT    165
CK2b_Rattus         QQGDFGYCPRVYCENQPMLPIGLSDIPGEAMVKLYCPKCMDVYTPKSSRHHHTDGAYFGT    161
CK2_H_sapiens       QQGDFGYCPRVYCENQPMLPIGLSDIPGEAMVKLYCPKCMDVYTPKSSRHHHTDGAYFGT    161
                     : :** **** * .: :**:* **:     ***:**:* *:* *:* **   ***::**

CK2b_T_cruzi        TFPHLFLMHLRENGKVISKPKQHYVPRIYGFRLREKGDAKEEHDDQQIAARVETEETEER    240
CK2b_Leishmania     TFPHLLLMQLRERGVSIPPPNQQYVPKVYGFRVRKPGTPLITNGAGEEDTGAA----SAG    230
CK2b_S_pombe        SFPHMLFQVYPEL--AVPKSQERYIPRIFGFKVHSYSATFKKQDVYKEKQKKRLQGAEAE    223
CK2b_Rattus         GFPHMLFMVHPEY--RPKRPANQFVPRLYGFKIHPMAYQLQLQAASNFKSPVKTIR----    215
CK2_H_sapiens       GFPHMLFMVHPEY--RPKRPANQFVPRLYGFKIHPMAYQLQLQAASNFKSPVKTIR----    215
                     ***:::    *         ::::*:::**:::  .     :   :

CK2b_T_cruzi        AAEVSVGESNTPANT---------PNNSRLARDCFRDPNGERAEAPQR    279
CK2b_Leishmania     AGDVSRKAEAGPHHKEEGAVAMSNTLDQRLSAESSSSSSKGKAE----    274
CK2b_S_pombe        S-----------KNK----LAIT-------------------------    231
CK2b_Rattus         ------------------------------------------------    215
CK2_H_sapiens       ------------------------------------------------    215
```

Figure S3: Multiple sequence alignment of CK2β orthologous from different species as indicated. Sequences were aligned using the ClustalW tool (https://www.genome.jp/tools-bin/clustalw). *S. pombe* CK2β possess four CK2α target serine residues at the first 14 aminoacid residues at the N-terminus domain of the polypeptide. Those serine residues are underlined.
