# Supplementary material for: T. cruzi DNA polymerase beta (Tcpolβ) is phosphorylated in vitro by CK1, CK2 and TcAUK1 leading to the potentiation of its DNA synthesis activity
Source: PLoS Negl Trop Dis. 2021 Jul 14;15(7):e0009588. doi: 10.1371/journal.pntd.0009588 (PMC8312956; doi:10.1371/journal.pntd.0009588)
Supplement: S4 Fig — Sequences were aligned using the Clustal Omega tool (https://www.ebi.ac.uk/Tools/msa/clustalo/). The active site and the activation loop of the enzyme from those species is indicated. (PDF) [file pntd.0009588.s004.pdf]

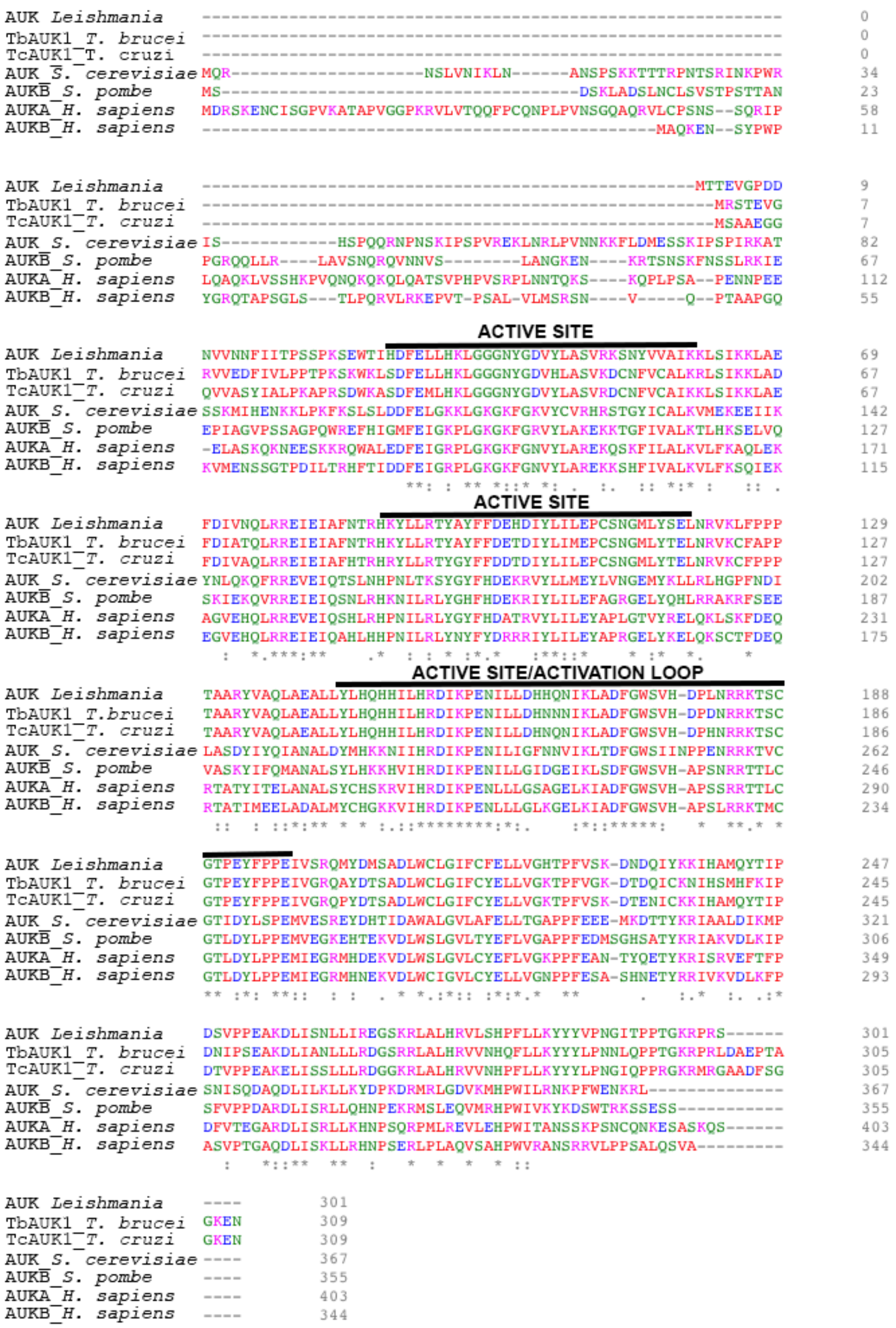


**Figure S4: Multiple sequence alignment of Aurora Kinase (AUK) orthologous from the indicated species.** Sequences were aligned using the Clustal Omega tool (https://www.ebi.ac.uk/Tools/msa/clustalo/). The active site and the activation loop of the enzyme from those species is indicated.
