# Supplementary material for: T. cruzi DNA polymerase beta (Tcpolβ) is phosphorylated in vitro by CK1, CK2 and TcAUK1 leading to the potentiation of its DNA synthesis activity
Source: PLoS Negl Trop Dis. 2021 Jul 14;15(7):e0009588. doi: 10.1371/journal.pntd.0009588 (PMC8312956; doi:10.1371/journal.pntd.0009588)
Supplement: S5 Fig — All sequences from T. cruzi containing shared regions with CK1 were aligned and showed in the figure. The alignment was generated using the NCBI Multiple Sequence Alignment Viewer using the information of the Genbank database. CK1 kinase studied in this work is highlighted in yellow and indicated with a red asterisk. Red: Highly conserved regions. Gray: Middle conserved regions. Blue: Low conserved regions. (PDF) [file pntd.0009588.s005.pdf]

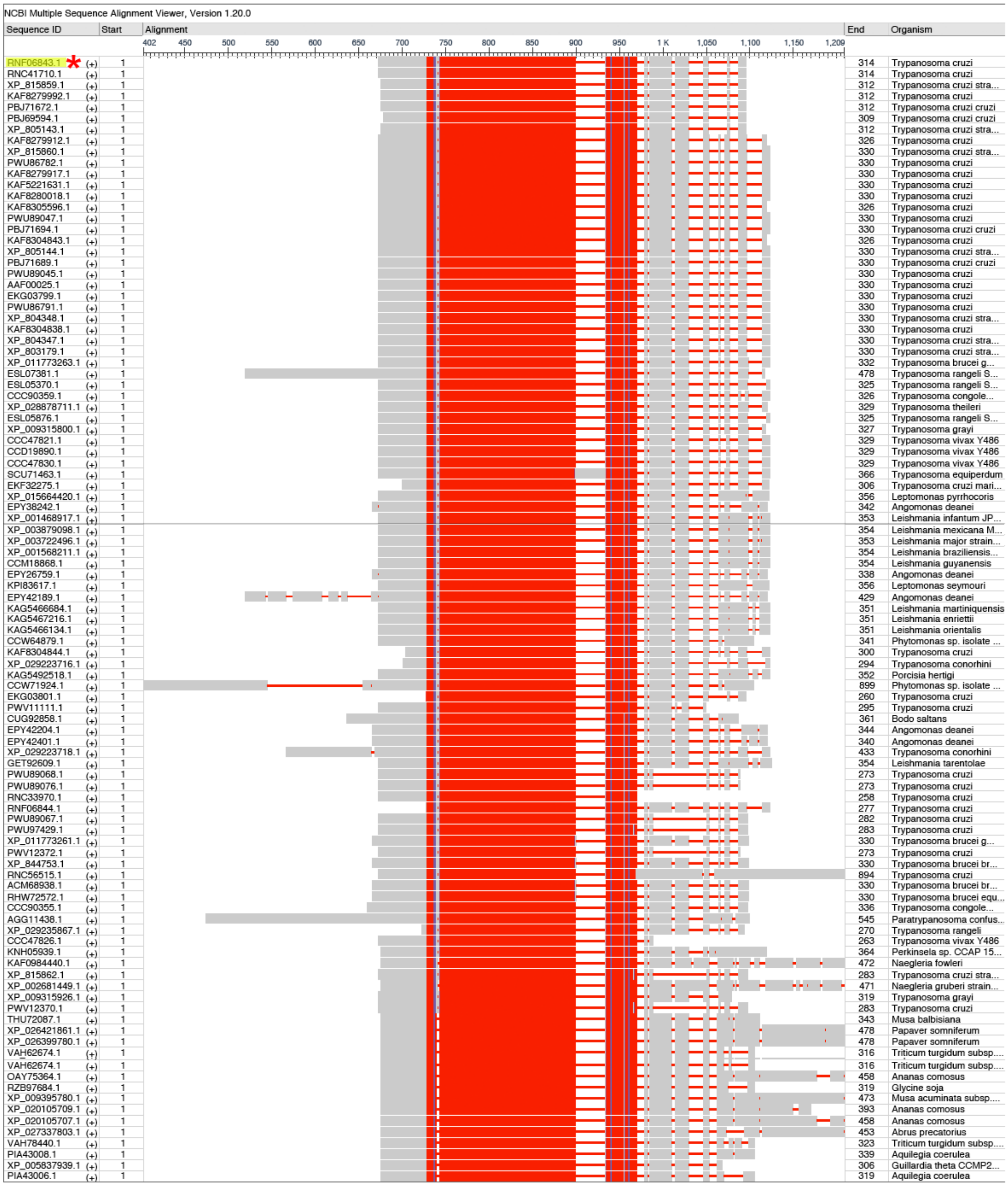

Figure S5: Multiple sequence alignment of CK1 protein kinases genes. All sequences from *T. cruzi* containing shared regions with CK1 were aligned and showed in the figure. The alignment was generated using the NCBI Multiple Sequence Alignment Viewer using the information of the Genbank database. CK1 kinase studied in this work is highlighted in yellow and indicated with a red asterisk. Red: Highly conserved regions. Gray: Middle conserved regions. Blue: Low conserved regions.
