# Supplementary material for: T. cruzi DNA polymerase beta (Tcpolβ) is phosphorylated in vitro by CK1, CK2 and TcAUK1 leading to the potentiation of its DNA synthesis activity
Source: PLoS Negl Trop Dis. 2021 Jul 14;15(7):e0009588. doi: 10.1371/journal.pntd.0009588 (PMC8312956; doi:10.1371/journal.pntd.0009588)
Supplement: S6 Fig — Amounts of 50, 100 and 200 ng of inactive polymerase were phosphorylated with 20 pmol of CK1α. It can be observed that unfolded Tcpolβ is not a substrate for the protein kinase. In the bottom of the figure it can be seen a coomassie blue stained SDS-PAGE with the different amounts of the analyzed proteins. (+) indicates control experiment with only 20 pmol of the protein kinase. (PDF) [file pntd.0009588.s006.pdf]

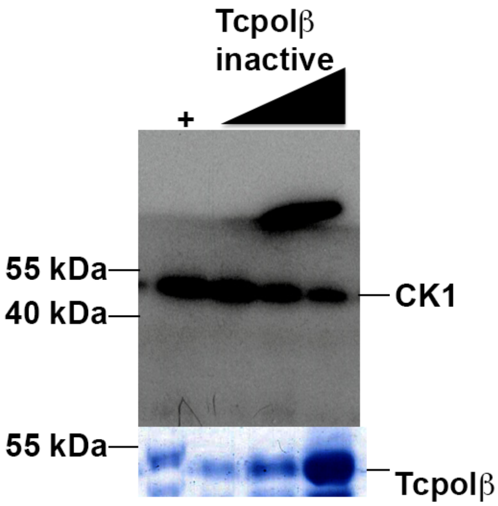

Figure S6. Unfolded inactive Tcpolβ is not a substrate for CK1α. Amounts of 50, 100 and 200 ng of inactive polymerase were phosphorylated with 20 pmol of CK1α. It can be observed that unfolded Tcpolβ is not a substrate for the protein kinase. In the bottom of the figure it can be seen a coomassie blue stained SDS-PAGE with the different amounts of the analyzed proteins. (+) indicates control experiment with only 20 pmol of the protein kinase.
