# Supplementary material for: T. cruzi DNA polymerase beta (Tcpolβ) is phosphorylated in vitro by CK1, CK2 and TcAUK1 leading to the potentiation of its DNA synthesis activity
Source: PLoS Negl Trop Dis. 2021 Jul 14;15(7):e0009588. doi: 10.1371/journal.pntd.0009588 (PMC8312956; doi:10.1371/journal.pntd.0009588)
Supplement: S7 Fig — Amounts of 50 and 100 ng of inactive Tcpolβ were assayed with 20 pmol of TcAUK1. It can be observed that AURKA does not phosphorylate unfolded Tcpolβ. A SDS-PAGE analysis is shown in the bottom of the figure. (-) indicates a negative control without protein kinases. (+) indicates a positive control with only 20 pmol of AURK. (PDF) [file pntd.0009588.s007.pdf]

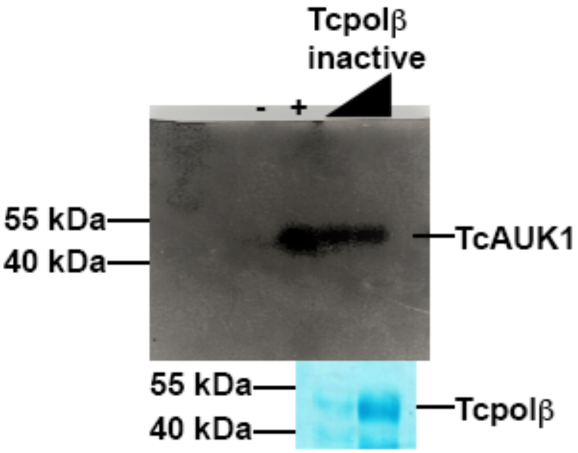

Figure S7. Unfolded Tcpolβ is not a substrate for TcAUK1. Amounts of 50 and 100 ng of inactive Tcpolβ were assayed with 20 pmol of TcAUK1. It can be observed that AURKA does not phosphorylate unfolded Tcpolβ. A SDS-PAGE analysis is shown in the bottom of the figure. (-) indicate a negative control without protein kinases. (+) indicates a positive control with only 20 pmol of AURK.
