# Supplementary material for: T. cruzi DNA polymerase beta (Tcpolβ) is phosphorylated in vitro by CK1, CK2 and TcAUK1 leading to the potentiation of its DNA synthesis activity
Source: PLoS Negl Trop Dis. 2021 Jul 14;15(7):e0009588. doi: 10.1371/journal.pntd.0009588 (PMC8312956; doi:10.1371/journal.pntd.0009588)
Supplement: S8 Fig — As indicated on the top of the figure, 15 pmol of S. pombe CK2β (pCK2β)(pCK2becated on the tα were used in the assays. It can be observed that TcCK2α does not get auto-phosphorylated, however, it can phosphorylate pCK2β, which has not activity by itself. (PDF) [file pntd.0009588.s008.pdf]

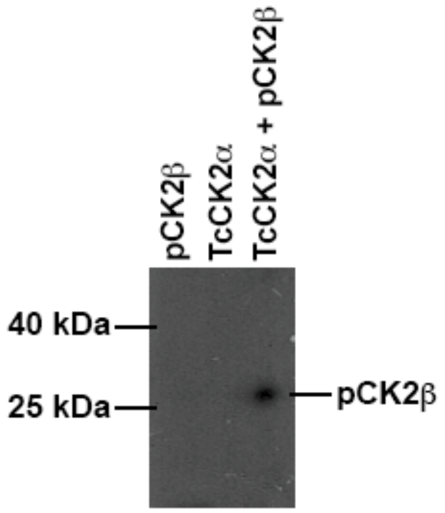


**Figure S8.** ***T. cruzi*** **CK2α does not get auto-phosphorylated.** As indicated on the top of the figure, 15 pmol of *S. pombe* CK2β (pCK2β) and 20 pmol of TcCK2α were used in the assays. It can be observed that TcCK2α does not get auto-phosphorylated, however, it can phosphorylate pCK2β, which has not activity by itself.
