# Supplementary material for: T. cruzi DNA polymerase beta (Tcpolβ) is phosphorylated in vitro by CK1, CK2 and TcAUK1 leading to the potentiation of its DNA synthesis activity
Source: PLoS Negl Trop Dis. 2021 Jul 14;15(7):e0009588. doi: 10.1371/journal.pntd.0009588 (PMC8312956; doi:10.1371/journal.pntd.0009588)
Supplement: Attachment — Submitted filename: CoverRespuestasPNTD.2021.docx [file pntd.0009588.s010.docx]

Peter J. Hotez

Editor-in-Chief

*PLOS Neglected Tropical Diseases*

1160 Battery Street

Koshland Building East, Suite 225

San Francisco, CA 94111

United States

Dear Dr. Hotez:

We would like to thank for the opportunity to re-submit our manuscript. We hope that the improvements of text and figures, as suggested by the reviewers, could allow the publication of the manuscript in PLOS Neglected Tropical Diseases. Below, you can find a detailed list of our responses to the reviewer comments and we have also submitted the manuscript with highlighted changes.

***Hypothesis***

**Reviewer 1.** We have deleted our hypothesis from the Author Summary and we propose an initial hypothesis on ROS signal transduction, according to the data that we are aware. This initial hypothesis is presented in the Discussion section and a schematic representation is presented in Figure 7.

***Methods***

**Reviewer 2.** A better explanation on the use of guanidine hydrochloride and the dialysis method for protein renaturation is given in Materials and Methods. It should be noted that recombinant protein kinases are produced as insoluble inclusion bodies that must be solubilized by using chaotropic agents, such as guanidine hydrochloride, which must be eliminated by dialysis to allow proper protein folding.

***Results***

**Reviewer 1.** We have modified the text to make a logical description of the results according to the figure numbers. Casein is a poor substrate, however, is clear from the figure that is a substrate for *T. cruzi* CK1α. The units are defined by the ImageJ program and a better explanation about the definition of units is given in Materials and Methods. It should be noted that in all quantifications, the same units and procedures were used.

We understand that the section describing *T. cruzi* CK2 might be a little bit confusing, however, we have eliminated any text that might appear confusing. The reason that we used pCK2β as a substrate and as stimulatory subunit is because we know from our earlier studies that this subunit is a good CK2 substrate and has stimulatory activity on CK2α. We believed that directly testing Tcpolβ as substrate, without phosphorylating a known CK2 substrate is a rather weak demonstration of CK2 activity. Likewise, TBB is a well characterized CK2 inhibitor, though it is unable to inhibit Tcpolβ phosphorylation, it is quite active inhibiting pCK2β phosphorylation (see Fig. 3D), suggesting that pCK2β phosphorylation is not artifactual as it is demonstrated in Fig S7.

Regarding “Effect of pharmacological inhibitors…”, we have modified the text and presented the results discussing first Fig. 3B and then Fig. 5 in a logical order.

**Reviewer 2.** In the text of this version, we have explained the minor lower band nature and we presented evidence that the increasing of the signal is not due to autophosphorylation of CK1α, since the amount of the enzyme remains constant and only varies the substrate. The signal of minor contaminating band does not augment by increasing the Tcpolβ substrate, therefore, it should be discarded from the analysis. Furthermore, we have included Figs. S6 that demonstrate that unfolded Tcpolβ is not a kinase substrate and increasing its amount does not increase the phosphorylation signal. Same criteria can be applied to phosphorylation of Tcpolβ by AURKA. We believe that the observation that unfolded Tcpolβ is not a good substrate for protein kinases, though interesting, escapes the scope of the manuscript.

Former Fig. S1 (actual Fig. S5) was corrected according to the reviewer suggestion. Former Fig. S4 (actual Fig. S3) correspond to a Clustal W analysis of CK2β subunits and those are stimulatory subunits and do not possess active site or activation loops.

TBB use is explained in the text. We have included Fig. S8 to demonstrate and explain that AURKA can phosphorylate Tcpolβ in a specific manner, since unfolded substrate does not produce any signal (Fig. S8). Signal quantification is explained in Material and Methods.

***Conclusions***

**Reviewer 1.** We have improved the presentation of Results and Materials and Methods sections according to reviewer suggestions.

**Reviewer 1.** We have improved the text grammar by using an online grammar and editing program. However, the reviewer must be aware that English is written and spoken in several styles and we have used the American English style. Moreover, we corrected the spelling and grammar by using the [Free, Powerful English Grammar Checker | SCRIBENS](https://www.scribens.com/) at https://www.scribens.com.

Reviewer 2. We added the verb “is” in that paragraph.

***General comments***

**Reviewer 1*.*** We agree that quantitative kinetic analysis it would be better to determine specific activity of the enzyme. However, it should be noted that we set out an assay in which a fixed amount of enzyme does not have basal detectable activity and increasing the amount of phosphorylating kinase improves in several fold the activity of the Tcpolβ, indicating that the phosphorylation event is the responsible of the high DNA synthesis by the polymerase. This observation is in complete agreement with those observed in *T. cruzi* cell extracts (H form) and cited in the manuscript.

**Reviewer 2.** We have improved the text according to the reviewer observations and we have not made any conclusions from our experiments, other than those supported by the presented data.

Sincerely yours

Dr. Edio Maldonado
