# Supplementary material for: T. cruzi DNA polymerase beta (Tcpolβ) is phosphorylated in vitro by CK1, CK2 and TcAUK1 leading to the potentiation of its DNA synthesis activity
Source: PLoS Negl Trop Dis. 2021 Jul 14;15(7):e0009588. doi: 10.1371/journal.pntd.0009588 (PMC8312956; doi:10.1371/journal.pntd.0009588)
Supplement: Attachment — Submitted filename: CoverRespuestasPNTD V2.2021.docx [file pntd.0009588.s011.docx]

Peter J. Hotez

Editor-in-Chief

*PLOS Neglected Tropical Diseases*

1160 Battery Street

Koshland Building East, Suite 225

San Francisco, CA 94111

United States

Dear Dr. Hotez:

First at all, we thank for the opportunity to re-submit a revised version of our manuscript. Second, we acknowledge to Reviewer 1 for useful comments on the manuscript that will certainly improve its academic quality. We have modified some sections of the manuscript according to the Reviewer 1 suggestions and we highlighted those changes.

1. We have added an extensive paragraph with available evidence on the role and importance of protein kinases in the signal transduction of trypanosomatids. We have added three additional references, namely 41,42 and 43 to support those statements. We think that our hypothesis on the role of CK1, CK2 and AURK in regulating the activity of Tcpolβ is easily testable by using classic pharmacological approaches, such as the use of known inhibitors or by using genetic approaches to assay gene function, as for example RNA interference to knockdown gene expression for those kinases encoded by a single gene or a knockout by using CRISP/Cas-9 approaches, and then analyze the altered biochemical processes. Also, a classic and direct approach is gene overexpression to analyze the phenotype of the gene-overexpressing strain.
2. In Fig.2 we showed that CK1α can phosphorylate casein and this phosphorylation is inhibited by heparin and we also showed that Tcpolβ is a substrate for CK1α as predicted in Fig.1. We did not want to move the figure of heparin inhibition to Fig. 5, since Fig. 5 is already large enough. CK2β subunits do not have catalytic activity, as they can only regulate the activity of the CK2α subunits. As stated, the inclusion of the *S. pombe* CK2β subunit to form a hybrid holoenzyme has three main purposes, which are: i) To stimulate the TcCK2α activity, ii) It serves as a substrate for TcCK2α, and iii) It is useful to test the effect of the specific mammalian CK2 inhibitor TBB. Furthermore, as suggested by the reviewer and stated, underscores the functional and structural conservation of those CK2β subunits through the evolution. Since, we used the *S. pombe* CK2β subunit to test the TBB inhibitory effect, we moved that figure from Fig. 3 to Fig. 5. In Fig.4 we showed that Tcpolβ is a substrate for AURKA as predicted in Fig.1. We have considered the possibility that the protein doublet might be two species of Tcpolβ phosphorylation, as this DNA polymerase has two phosphorylation consensus sites. Nevertheless, Tcpolβ is a substrate for AURKA. In Fig.6, the assays used to analyze Tcpolβ activation by the three kinases are indeed qualitative ones, as stated.
3. As indicated in point 1, we have added a body of information regarding protein kinases in trypanosomatids to support our hypothesis. As stated, we have recently published a paper (reference 19) that shows that a heavily phosphorylated Tcpolβ (H form) is active in DNA synthesis, whereas a dephosphorylated L form is less active in DNA synthesis. This, together with the actual results of the presented manuscript suggest that CK2, CK1 and AURKA might be involved in a signal transduction cascade, an easily testable hypothesis, as stated in point 1 of this letter.

Sincerely yours

Dr. Edio Maldonado
