# Supplementary material for: T. cruzi DNA polymerase beta (Tcpolβ) is phosphorylated in vitro by CK1, CK2 and TcAUK1 leading to the potentiation of its DNA synthesis activity
Source: PLoS Negl Trop Dis. 2021 Jul 14;15(7):e0009588. doi: 10.1371/journal.pntd.0009588 (PMC8312956; doi:10.1371/journal.pntd.0009588)
Supplement: Attachment — Submitted filename: CoverRespuestasPNTD V3.2021.docx [file pntd.0009588.s012.docx]

Ziyin Li, PhD.

Guest Editor

*PLOS Neglected Tropical Diseases*

Dear Dr. Li:

Thank you very much for the observation about the *T. cruzi* gene that we called AURKA. We agree with you that gene is the functional orthologue of AURKB considering its size product, location and function. We have changed its name to TcAUK1, since it is closely related to TbAUK1 and to avoid any confusion in the readers. Although, we had discussed at the end of the Discussion section that our named AURKA gene was identical to the previously described TcAUK1 by Fassolari *et al.* We expanded a little bit that part of the paragraph in the manuscript Discussion section, since those authors found at least three AURK homologue genes in *T. cruzi*. However, a phylogenetic analysis indicates that TcAUK1 is closer to human AURKA than to AURKB, but that is a matter that escapes this analysis. Accordingly, we have compared TcAUK1 with TbAUK1 and other AURKB orthologues, as indicated in Fig. S4. We hope that the revision makes the manuscript suitable for publication in *PLOS Neglected Tropical Diseases*.

Sincerely yours

Dr. Edio Maldonado
